# Reduced Peripheral Expression of the Glucocorticoid Receptor α Isoform in Individuals with Posttraumatic Stress Disorder: A Cumulative Effect of Trauma Burden

**DOI:** 10.1371/journal.pone.0086333

**Published:** 2014-01-21

**Authors:** Hannah Gola, Andrea Engler, Julia Morath, Hannah Adenauer, Thomas Elbert, Iris-Tatjana Kolassa, Harald Engler

**Affiliations:** 1 Clinical and Biological Psychology, University of Ulm, Ulm, Germany; 2 Institute of Medical Psychology and Behavioral Immunobiology, University Hospital Essen, University of Duisburg-Essen, Essen, Germany; 3 Clinical Psychology and Neuropsychology, University of Konstanz, Konstanz, Germany; University of Wuerzburg, Germany

## Abstract

**Background:**

Posttraumatic stress disorder (PTSD) is a serious psychiatric condition that was found to be associated with altered functioning of the hypothalamic-pituitary-adrenal (HPA) axis and changes in glucocorticoid (GC) responsiveness. The physiological actions of GCs are primarily mediated through GC receptors (GR) of which isoforms with different biological activities exist. This study aimed to investigate whether trauma-experience and/or PTSD are associated with altered expression of GR splice variants.

**Methods:**

GRα and GRβ mRNA expression levels were determined by real-time quantitative PCR in whole blood samples of individuals with chronic and severe forms of PTSD (*n = *42) as well as in ethnically matched reference subjects (non-PTSD, *n = *35).

**Results:**

Individuals suffering from PTSD exhibited significantly lower expression of the predominant and functionally active GRα isoform compared to non-PTSD subjects. This effect remained significant when accounting for gender, smoking, psychotropic medication or comorbid depression. Moreover, the GRα expression level was significantly negatively correlated with the number of traumatic event types experienced, both in the whole sample and within the PTSD patient group. Expression of the less abundant and non-ligand binding GRβ isoform was comparable between patient and reference groups.

**Conclusions:**

Reduced expression of the functionally active GRα isoform in peripheral blood cells of individuals with PTSD seems to be a cumulative effect of trauma burden rather than a specific feature of PTSD since non-PTSD subjects with high trauma load showed an intermediate phenotype between PTSD patients and individuals with no or few traumatic experiences.

## Introduction

Posttraumatic stress disorder (PTSD) is a serious psychiatric condition that can develop in the aftermath of traumatic events such as life-threatening accidents, natural disasters, physical assaults, sexual abuse, combat experience or torture. Characteristic symptoms of PTSD include re-experiencing of the traumatic event in the form of intrusive recollections, flashbacks or nightmares; persistent avoidance of stimuli associated with the traumatic event; emotional numbing; as well as a constant state of heightened alertness and increased arousal [1]. In addition to psychiatric morbidity, PTSD appears to be associated with altered functioning of the hypothalamic-pituitary-adrenal (HPA) axis and changes in central and/or peripheral glucocorticoid (GC) responsiveness [2]–[5], but the underlying mechanisms are not sufficiently understood yet.

Glucocorticoids exert their diverse actions primarily through cytoplasmic GC receptors (GRs) that are ubiquitously expressed in many tissues and cell types. Upon hormone binding, the activated GR translocates to the nucleus and acts as a ligand-dependent transcription factor that modulates the expression of GC-responsive genes or modifies the activity of other transcription factors such as NF-κB or AP-1 [6], [7]. In humans, two highly homologous isoforms of the GR have been identified [8], which are derived from the same gene by alternative splicing of the primary transcript [9], [10]. *GRα* (777 amino-acid protein), the predominant and functionally active receptor, is generated through splicing of exon 8 to the proximal part of exon 9 (9α), whereas *GRβ* (742 amino-acid protein) is created through splicing of exon 8 to the distal part of exon 9 (9β). The two receptor isoforms share identical *N*-termini encoded by exons 2–8 (727 amino acids), but GRβ differs from GRα in its *C*-terminus, where the 50 amino acids derived from exon 9α are replaced by a 15 amino-acid region encoded by exon 9β. Since this region contains important parts of the ligand binding domain, GRβ is unable to bind natural and synthetic GCs such as cortisol and dexamethasone, respectively [11], [12]. In addition, no transcriptional activation or repression activities in response to hormone were found. Instead, it has been reported that GRβ might act as a dominant-negative inhibitor of GRα, either through competition for co-regulators or through formation of inactive GRα/GRβ heterodimers [13], [14]. Because of the ability of GRβ to negatively regulate the action of GRα, it has been suggested that the ratio of GRα/GRβ expression might be a critical factor determining the GC responsiveness of target tissues.

Alterations in central and peripheral expression of GR splice variants have been reported for individuals with mood/affective disorders [15], [16]. For example, GRα mRNA expression was shown to be reduced in peripheral blood cells of bipolar and major depressive disorder (MDD) patients during depressive state as well as in remission compared to healthy controls, while expression of the GRβ splice variant was not altered [16]. In PTSD patients, expression of GR isoforms has not been investigated yet, although total GR gene expression, GR number, and GC binding capacity were found to be altered in individuals suffering from this disorder [17]–[21].

The aim of this study was to investigate whether trauma-experience and/or PTSD are associated with changes in the expression of GR splice variants. GRα and GRβ mRNA expression levels were determined by real-time quantitative PCR in whole blood samples of individuals with war- and torture-related PTSD as well as in non-PTSD subjects with low and high levels of trauma exposure.

## Methods

### Ethics Statement

All procedures were approved by the Ethics Committee of the University of Konstanz and were carried out in accordance with the Declaration of Helsinki (2008). Written informed consent was obtained from all individuals enrolled in the study.

### Participants

Forty-two individuals with current PTSD (21 male, 21 female; mean age = 32.6 years, SD = 9.1, range 17–51) according to the DSM-IV and 35 non-PTSD subjects (13 male, 22 female; mean age = 29.1 years, SD = 9.7, range 17–61) were included in this study. Twenty of the subjects (11 PTSD, 9 non-PTSD) had already participated in a previous study on inflammatory markers (22). PTSD patients were refugees with chronic (mean symptom duration = 8.1 years, SD = 5.7) and severe (mean sum score in the Clinician Administered PTSD Scale [CAPS] = 84.2, SD = 17.3) forms of PTSD due to multiple, highly stressful war and torture experiences. In addition to the PTSD diagnosis, 33 patients (78.6%) met the DSM-IV criteria for a current major depressive episode. Sixteen PTSD patients (38.1%) reported regular intake of psychotropic medication and four women reported the use of oral contraceptives (Table 1). Twelve PTSD patients (28.6%) were smokers. All patients were recruited from the Psychotrauma Research and Outpatient Clinic for Refugees, University of Konstanz, Germany.

**Table 1:** Sociodemographic and clinical characteristics.

Variable	non-PTSD(*n* = 35)	PTSD(*n* = 42)	*p*
Age^a^ (years)	29.1±9.7	32.6±9.1	0.11
Gender (Female/Male)	22/13	21/21	0.26
Region of origin (%)			0.59
* Africa*	28.6	28.6	
* Balkan*	17.1	9.5	
* Middle East/Afghanistan*	54.3	61.9	
Psychotropicmedication (%)			<0.001
* Hypnotics*	0	14.3	
* Anxiolytics*	0	11.9	
* Antidepressants*	0	28.6	
* Neuroleptics*	0	7.1	
Contraceptives (%)	17.1	9.5	0.64
Smoking (%)	14.3	28.6	0.13
* Cigarettes per day* a	0.8±2.4	5.7±11.4	<0.01
Number of traumaticevent types^a^			
* War or torture events*	1.8±4.4	10.4±5.8	<0.001
* CAPS events*	3.5±2.3	7.4±2.0	<0.001
CAPS score^a^	3.7±8.0	84.2±17.3	<0.001
HAM-D score^a^	2.9±3.5	27.1±8.3	<0.001
SOMS-7 score^a^	5.0±6.7	29.2±12.5	<0.001

a Data are presented as mean ± SD. Group differences were analyzed using chi-square tests (categorical data) and *t*-tests (continuous data). CAPS, Clinician Administered PTSD Scale; HAM-D, Hamilton Depression Rating Scale; SOMS-7, Screening for Somatoform Symptoms-7.

The non-PTSD comparison group was recruited through advertisement and was matched to the patient group with regard to age and region of origin (see Table 1). Except for six women reporting the intake of oral contraceptives, all reference subjects were free of medication. Five non-PTSD subjects (14.3%) were smokers. Since the non-PTSD group varied with respect to the number of traumatic event types experienced (range: 0–8), some of the analyses were repeated with a three-group design. For this purpose, we divided the non-PTSD comparison group by median split into a group with substantial exposure to traumatic stressors (4–8 different traumatic event types; *high trauma-exposed non-PTSD*) and a group with no or few traumatic experiences (0–3 traumatic event types; *low trauma-exposed non-PTSD*) based on the number of past traumatic event types assessed with the event checklist of the CAPS.

Exclusion criteria for the study were intake of glucocorticoids or acute and chronic somatic illnesses. In addition, non-PTSD subjects were excluded if they met the criteria for any mental disorder according to DSM-IV, whereas PTSD patients were excluded if they met the criteria for comorbid alcohol and substance abuse or dependence, or a current or past history of psychosis according to the DSM-IV.

### Clinical Interviews

All participants underwent an extensive standardized clinical interview administered by experienced clinical psychologists and trained translators. PTSD symptoms and the number of traumatic event types experienced were assessed with the CAPS [23]. The vivo Checklist of War, Detention and Torture events [24], which assesses common traumatic experiences in conflict regions and during torture, allowed for a detailed evaluation of the number of traumatic event types experienced. The Mini International Neuropsychiatric Interview (M.I.N.I.) was used to screen for potential comorbid mental disorders [25]. In addition, the severity of depressive symptoms was assessed with the Hamilton Depression Rating Scale (HAM-D; [26]). The Screening for Somatoform Symptoms-7 (SOMS-7) was used to check for potential somatoform disorders [27]. After complete description of the study to the subjects, written informed consent was obtained.

### Specimen Collection

Blood samples were obtained from antecubital veins between 10 a.m. and 11 a.m., before starting with the clinical interviews. Blood for total RNA extraction was collected in PAXgene Blood RNA Tubes (PreAnalytiX GmbH, Hombrechtikon, Switzerland) and was, after an initial freezing step at –20°C for 24 h, stored at –80°C until further processing. Blood for plasma cortisol determination and complete blood cell (CBC) analysis was drawn into EDTA-treated tubes (BD Vacutainer, Franklin Lakes, NY, USA). Plasma was separated by centrifugation (3000×g, 10 min, 4°C) and stored at –28°C.

### GR Isoform mRNA Expression

PAXgene Blood RNA Tubes were thawed at room temperature and were incubated for 2 h after reaching ambient temperature. Total RNA was isolated from whole blood samples using the PAXgene Blood RNA Kit (Qiagen, Hilden, Germany) according to the manufacturer’s instructions. RNA concentration and purity were determined photometrically using a LabelGuard Microliter Cell (Implen, Munich, Germany) together with a BioPhotometer (Eppendorf, Hamburg, Germany). The A_260_/A_280_ ratios of all samples were within the recommended range between 1.8 and 2.1. First-strand cDNA was synthesized from 1.5 µg total RNA using the High Capacity cDNA Reverse Transcription Kit (Applied Biosystems, Darmstadt, Germany). Real-time quantitative PCR was performed on a 7500 Fast Real-Time PCR System (Applied Biosystems) using FAST qPCR MasterMix Plus Low ROX (Eurogentec, Seraing, Belgium) and the following cycling conditions: 5 min at 95°C followed by 50 cycles of 15 s at 95°C, 30 s at 60°C and 30 s at 72°C. Primers and probes were adapted from [28] and purchased from Microsynth (Balgach, Switzerland). A common forward primer (5′-TGTTTTGCTCCTGATCTGA-3′), located in exon 6, as well as a common probe (5′-6-FAM-TGACTCTACCCTGCATGTACGAC-BHQ1–3′), located in exon 7, were used for both GR splice variants. To discriminate among GR isoforms, specific reverse primers for GRα (5′-TCGGGGAATTCAATACTCA-3′), located in exon 9α, and GRβ (5′- TGAGCGCC-AAGATTGT-3′), located in exon 9β, were used. Hypoxanthine phosphoribosyl transferase (HPRT) served as endogenous reference gene (forward primer: 5′-CACTGGCAAAACAATGCAGACT-3′; reverse primer: 5′-GTCTGGCTTATATCCAACACTTCGT-3′; probe: 5′-6-FAM-CAAGCTTGCGACCTTGACCATCT-TTGGA-BHQ1–3′). For quantification of gene expression, serially diluted cDNA samples generated from purified specific PCR products (High Pure PCR Product Purification Kit, Roche Diagnostics, Mannheim, Germany) were used as external standards in each run. Results are expressed as fg/µg total RNA.

### Complete Blood Count

A complete blood count (CBC) including white blood cell differential was obtained using an automated hematology analyzer (XT-2000i, Sysmex, Horgen, Switzerland).

### Cortisol Analysis

Total plasma cortisol concentrations were measured using a commercial enzyme-linked immunosorbent assay (Cortisol ELISA, IBL International, Hamburg, Germany) according to the manufacturer’s instructions. Intra- and interassay variances were 5.6% and 6.9%, respectively.

### Statistical Analysis

Data analysis was performed using SPSS 17.0 (SPSS Inc., Chicago, IL, USA). The level of significance was set at *p*<0.05. Normality of residuals was examined using the Kolmogorov-Smirnov test and data were log-transformed when necessary. Non-transformed data are presented in figures and tables. Group comparisons between non-PTSD subjects and PTSD patients were performed applying chi-square tests (categorical data) and *t*-tests (continuous data). For multiple group comparisons, data were analyzed by analysis of variance (ANOVA). Tukey’s HSD test was used for post hoc comparisons. Spearman’s rank correlation coefficient was applied to measure the strength of association between GR mRNA expression and clinical variables.

## Results

### Sociodemographic and Clinical Characteristics

PTSD (*n = *42) and non-PTSD groups (*n = *35) did not significantly differ in age, gender, region of origin, and intake of contraceptives (Table 1). However, PTSD patients smoked significantly more cigarettes per day than non-PTSD subjects. With respect to clinical variables, the PTSD group had experienced significantly more traumatic event types than the comparison group and had significantly higher CAPS, HAM-D, and SOMS-7 scores.

### Cellular Composition of the Blood

Since GRα and GRβ expression levels were shown to vary among leukocyte subpopulations [29], [30], we examined whether the cellular composition of the blood samples was comparable between PTSD patients and non-PTSD subjects. As shown in Table 2, total leukocyte numbers and percentages of leukocyte subsets did not significantly differ between study groups.

**Table 2:** Blood cell parameters and plasma cortisol concentration.

Variable	non-PTSD(*n* = 35)	PTSD(*n* = 42)	Statistics
Leukocytes (10^9^/l)	5.82±1.21	5.77±1.95	*t* = 0.13, *p* = 0.89
Lymphocytes (%)	34.9±8.2	34.5±7.9	*t* = 0.23, *p* = 0.82
Neutrophils (%)	53.4±9.3	53.7±10.5	*t* = −0.15, *p* = 0.88
Monocytes (%)	8.3±2.3	8.0±2.3	*t* = 0.67, *p* = 0.50
Eosinophils (%)	2.9±1.9	3.3±2.7	*t* = −0.84, *p* = 0.40
Basophils (%)	0.4±0.2	0.4±0.2	*t* = 0.47, *p* = 0.64
Cortisol (nmol/l)	345.4±172.7	308.6±142.4	*t* = 0.99, *p* = 0.32

Data are presented as mean ± SD.

### Expression of GRα and GRβ Isoforms

**Figure 1:**
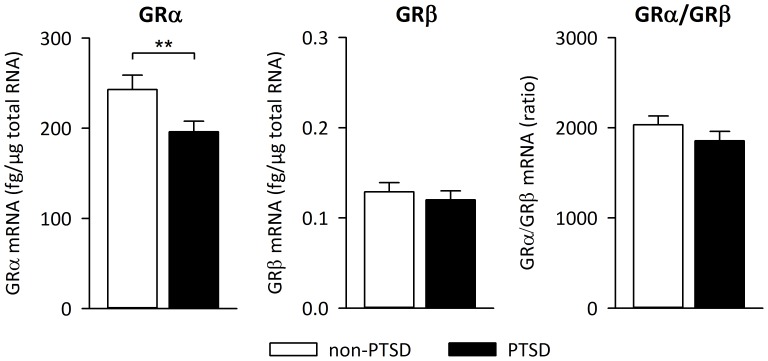
Glucocorticoid receptor isoform expression. Expression levels of glucocorticoid receptor (GR) α and β isoforms as well as GRα/GRβ ratio in the peripheral blood of non-PTSD subjects (*n* = 35) and PTSD patients (*n* = 42) measured by real-time quantitative PCR. Means and SEM are shown. ** *p* = 0.01.

GRα and GRβ mRNA was detectable in blood samples from all study participants. Independent of PTSD diagnosis, GRα mRNA levels were about 2,000-fold higher than GRβ mRNA levels. PTSD patients exhibited significantly lower GRα mRNA expression levels compared to the non-PTSD comparison group [*F*(1,75) = 6.89, *p* = 0.01] whereas expression of the GRβ isoform did not differ between groups [*F*(1,75) = 0.85, *p* = 0.36] (Fig. 1). In addition, no significant group difference was observed for the GRα/β ratio [*F*(1,75) = 2.56, *p* = 0.11]. Expression of HPRT, which served as reference gene, did not differ between PTSD patients and non-PTSD individuals [*F*(1,75) = 0.58, *p* = 0.45].

The above reported difference in GRα mRNA expression remained significant when ‘number of cigarettes per day’ [*F*(1,74) = 6.70, *p*<0.05] was introduced as covariate into the model. Moreover, the effect became even stronger when all participants with psychotropic medication were excluded from the statistical analysis [*F*(1,59) = 12.53, *p*<0.01]. In a next step, we repeated the analyses with ‘gender’ or ‘major depression’ as additional between-subject factors to account for the possible influence of these variables on the effect reported here. For the expression of both GRα and GRβ mRNA neither significant main effects of ‘gender’ nor significant ‘group × gender’ interactions could be identified. After introducing ‘gender’ as additional covariate, the significant group difference for the GRα remained statistically significant [*F*(1,73) = 8.33, *p*<0.01]. Regarding the influence of major depression on GR expression, we found a main effect of ‘depression’ on the expression of GRα [*F*(1,73) = 5.58, *p*<0.05]. Again, the effect of PTSD diagnosis on GRα expression remained statistically significant [*F*(1,73) = 13.34, *p*<0.001].

### GR Isoform Expression and Plasma Cortisol

To determine whether the observed group difference in GRα mRNA expression was related to individual differences in basal glucocorticoid secretion, we additionally measured blood cortisol levels in samples that were obtained at the same time as the mRNA samples. Plasma cortisol concentrations did not significantly differ between PTSD patients and non-PTSD subjects (Table 2). Furthermore, no significant correlations between plasma cortisol and GRα (*r = *0.09, *p* = 0.46) or GRβ (*r* = 0.09, *p* = 0.42) mRNA expression levels could be found.

### GR Isoform Expression and Clinical Variables

**Figure 2:**
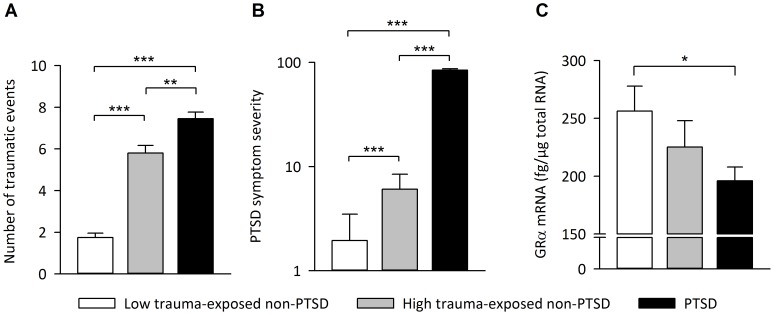
Relationship between trauma load and GRα isoform expression. (A) Number of different traumatic event types, (B) PTSD symptom severity, and (C) GRα mRNA expression levels in non-PTSD subjects with no or few traumatic experiences (0–3 traumatic event types; *low trauma-exposed non-PTSD*; *n* = 20), in non-PTSD subjects with substantial exposure to traumatic stressors (4–8 traumatic event types; *high trauma-exposed non-PTSD*; *n* = 15), and in PTSD patients (*n* = 42). Means and SEM are shown. * *p*<0.05, ** *p*<0.01, *** *p*<0.001.

To elucidate whether the observed reduction in GRα mRNA expression is a specific feature of PTSD or rather constitutes a general consequence of trauma exposure, we repeated the above reported analyses after subdividing the non-PTSD group into a group with no or few traumatic experiences (*low trauma-exposed; n* = 20) and a group with substantial exposure to traumatic stressors (*high trauma-exposed*; *n* = 15). With respect to the reduction in GRα mRNA expression, the high trauma-exposed non-PTSD group displayed an intermediate phenotype, positioned between PTSD patients and the low trauma-exposed non-PTSD group, indicating a cumulative effect of trauma exposure on GRα mRNA expression (Fig. 2). Furthermore, the number of traumatic event types assessed with the CAPS event list was significantly negatively correlated with GRα mRNA expression level (*r* = –0.29, *p*<0.01). *Within* the PTSD group, GRα mRNA expression was again negatively correlated with the number of traumatic event types experienced (*r* = –0.32, *p*<0.05). In addition, GRα mRNA expression was negatively correlated with the hyperarousal subscale of the CAPS (*r* = –0.37, *p*<0.05) but was neither related to the total CAPS score (*r* = –0.02, *p* = 0.88), nor to intrusions (*r = *0.02, *p* = 0.90) or avoidance symptoms (*r = *0.08, *p* = 0.62). Furthermore, GRα mRNA expression showed no significant correlations with the duration of PTSD symptoms (*r = *0.13, *p* = 0.40), HAM-D (*r* = –0.04, *p* = 0.80) or SOMS-7 scores (*r = *0.06, *p* = 0.71).

## Discussion

This study provides evidence for an altered GR isoform expression in individuals suffering from chronic and severe forms of PTSD. Peripheral blood mRNA expression of the predominant and functionally active GRα splice variant was about 20% lower in patients with war- and torture-related PTSD compared to non-PTSD control subjects without psychiatric diagnosis. In contrast, expression of the less abundant GRβ isoform, which does not bind GCs, was comparable between PTSD and non-PTSD groups. The results were neither influenced by the cellular composition of the blood samples nor by gender, smoking or psychotropic medication. In addition, GRα mRNA expression levels were not related to individual plasma cortisol concentrations, which did not significantly differ between PTSD and non-PTSD groups.

The observation of a diminished expression of the ligand-binding GRα isoform in severely traumatized PTSD patients is consistent with the findings of various studies that reported reduced GR numbers and/or GC binding capacity in blood immune cells of individuals with war-related PTSD [17]–[19], [21]. However, other reports found that GR numbers may actually be higher [31], [32] or similar [33], [34] in PTSD compared to reference subjects. This inconsistency in the findings may be related to variations in sample characteristics (e.g., type of trauma, time elapsed since the traumatic event, PTSD symptom severity, use of reference groups with or without history of trauma) and differences in the techniques used to estimate GR numbers (e.g., mRNA/protein expression, radioligand binding, flow cytometry).

A major risk factor for the development and persistence of PTSD constitutes the number of different traumatic event types experienced, with studies showing that higher trauma exposure is associated with higher prevalence of PTSD [35], [36] as well as with lower probability of spontaneous remission from PTSD [37]. Our data suggests that such a dose-response effect of trauma load exists not only at the level of psychiatric symptoms but also at the physiological level, as non-PTSD subjects with substantial trauma experience (4–8 traumatic event types) show an intermediate GRα expression level between low trauma-exposed non-PTSD subjects (0–3 traumatic event types) and PTSD patients. This assumption is further supported by the observation that the number of traumatic event types was negatively correlated with GRα expression levels in the whole sample as well as within the PTSD patient group.

PTSD is not the only psychiatric condition that can develop following exposure to traumatic events. More than three-quarter of individuals suffering from PTSD meet the criteria for at least one other psychiatric disorder, with major depression being the most prevalent condition occurring concurrently with PTSD [38]. The present study included a sample of severely traumatized subjects (victims of war and torture) of which 78.6% fulfilled the criteria for a current major depressive episode. Since reduced GRα mRNA expression as well as unchanged GRβ mRNA expression in peripheral blood cells were also found in individuals with major depressive disorder, both during the depressive episode and in remission [16], it is plausible to argue that comorbid depression might have contributed to the observed GR isoform expression pattern in PTSD patients. However, various etiological routes may lead to depression symptoms, trauma exposure being one of them [38], [39]. Since the inclusion of comorbid depression as an additional factor in our analysis did not eliminate the reported effect and GRα mRNA expression levels did not correlate with HAM-D scores, we assume that the observed group difference in GRα expression cannot be attributed to forms of depression unrelated to trauma exposure.

The reduced expression of the functionally active GRα isoform in the peripheral blood of PTSD subjects might be linked to the heightened inflammatory activity and immune dysregulation that has previously been found in subjects suffering from PTSD [22], [40]–[43]. For example, PTSD patients were reported to exhibit increased peripheral NF-κB pathway activity [44] as well as increased circulating levels of inflammatory markers such as interleukin (IL)-6 and C-reactive protein (CRP) [45], [46]. Moreover, these changes were reported to correlate with PTSD symptom severity [43], [44]. Since endogenous GCs restrain inflammatory responses, e.g., via repression of NF-κB-mediated activation of pro-inflammatory genes, it can be speculated whether the observed reduction in peripheral GRα expression contributes to low-grade inflammation in PTSD.

The current study has notable strengths such as the assessment of PTSD diagnosis by applying the CAPS as clinical interview by experienced experts and the inclusion of low and high trauma-exposed non-PTSD reference groups. Nevertheless some limitations should be considered as well. For instance, we did not assess GR isoform expression beyond the transcriptional level to determine whether the observed group differences in mRNA expression are also reflected at the protein level, even though earlier studies have shown that, in most human tissues including peripheral blood cells, GRα protein expression matches up quite well with GRα mRNA levels [28], [30]. Furthermore, the design of our study does not allow disentangling the effects of PTSD *per se* from the effects of trauma load, a problem which is, however, not easily solved since trauma exposure itself constitutes a well-described risk factor for the development of PTSD [35], [36]. The approach to collect a reference group matching the PTSD group with respect to the number of traumatic events experienced, sometimes considered as gold standard, is problematic itself (e.g., by recruiting an especially resilient comparison group), and was therefore not adopted in our study.

Taken together, this study provides novel evidence for reduced expression of the ligand-binding and functionally active GRα isoform in the blood of severely traumatized individuals, suggesting that the responsiveness of peripheral target cells towards GCs might be diminished in patients with chronic and severe forms of PTSD. However, GC sensitivity is not only determined by GR expression but also influenced by several other factors such as post-translational modifications, the composition of the GR complex, and the efficiency of the ligand-induced nuclear translocation of the GR [47], [48]. Thus, future studies should aim at elucidating whether important steps in the GR signaling cascade are additionally affected in severely traumatized subjects.
